# BRAF activating mutations involving the β3-αC loop in V600E-negative anaplastic pleomorphic xanthoastrocytoma

**DOI:** 10.1186/s40478-018-0525-1

**Published:** 2018-03-15

**Authors:** Drew Pratt, Sandra Camelo-Piragua, Kathryn McFadden, Denise Leung, Rajen Mody, Arul Chinnaiyan, Carl Koschmann, Sriram Venneti

**Affiliations:** 1 Department of Pathology, University of Michigan, Ann Arbor, MI 48104 USA; 2 Department of Neurology, University of Michigan, Ann Arbor, MI USA; 3 Department of Pediatrics, University of Michigan, Ann Arbor, MI USA; 4 Michigan Center for Translational Pathology, Ann Arbor, MI USA; 5 Department of Pediatrics, Division of Pediatric Hematology/Oncology, Mott Children’s Hospital at the University of Michigan, Ann Arbor, MI USA

Anaplastic pleomorphic xanthoastrocytoma (A-PXA, WHO grade III) is a newly defined entity with high-grade histopathologic features and a propensity for recurrence [6]. While PXA with low-grade histology (WHO grade II) can harbor a recurrent valine-to-glutamic acid (p.V600E) point mutation in BRAF in up to 78% of cases [6], the genomic drivers of A-PXA are poorly understood as the V600E mutation is absent in over half of A-PXAs [4]. Alterations reported to date in V600E-negative cases have included novel BRAF fusions and copy number alterations (Table 1).

**Table 1:** Genomic alterations in BRAF V600E-negative anaplastic pleomorphic xanthoastrocytoma

Reference^a^	Number of cases	Method	Alteration (s) (cases/total tested)	Clinical outcome (s)
Mistry et al., 2015	3	WES, aCGH	CDKN2A HD (1/3) TP53 mutation (1/2)	No individual case data available
Phillips et al., 2016	2	NGS	Case 1: NRF1-BRAF (1/2) Case 2: ATG7-RAF1 (1/2) CDKN2A HD (2/2)	Case 1: GTR with recurrence, f/u 48 months, deceased Case 2: GTR with recurrence, f/u 15 months, alive
Alexandrescu et al., 2016	1^b^	FISH, methylation analysis (450 k)	CDKN2A HD Gains: + 5, 7, 9q, 12p, 14q, 16q, 22q Losses: −1, 6, 13q, 14q, 21q	GTR, no recurrence, f/u 10 months, alive
Hsiao et al., 2017	1	WES, transcriptome	TMEM106B-BRAF	Resection, PFS 6 months, field radiation, contralateral recurrence, STR, progression, chemo with TMZ, stable and alive
Vaubel et al., 2017	6	Chromosomal microarray (OncoScan)	Gains: + 7 (3/6), + 5 (2/6) Losses: −22 (4/6), −14 (4/6), − 13 (3/6), − 10 (3/6), −1p (chromothripsis) CDKN2A HD (5/6)	No individual case data available
Korshunov et al., 2017	20 ^b,c^	Methylation analysis (450 k), targeted sequencing	TERT c.-124C > T (5/20); CDKN2A HD (8/20)	No individual case data available
Current study	2	WES, WGS, transcriptome	Case 1: BRAF p.L485_P490delinsF; FOXO1 p.A38T; HTR2A p.D48N; CDKN2A HD Case 2: BRAF p.V504_R506dup; KAT6A p.T1210 fs (subclonal) Gains (case 2): + 5, 6, 7, 10, 12, 15 Losses (case 1): −9, 22	Case 1: near-GTR, A9952 (carboplatin, vincristine), f/u 6 months, alive Case 2: subtotal resection, chemoradiation with TMZ, alive at last f/u 4 months post-dx

*WES* whole exome sequencing, *aCGH* array comparative genomic hybridization, *NGS* targeted next-generation sequencing, *IHC* immunohistochemistry, *FISH* fluorescence in situ hybridization, *WGS* whole genome sequencingm, *HD* homozygous deletion, *PM* promoter methylation, *GTR* gross total resection, *f/u* follow-up, *PFS* progression-free survival, *STR* stereotactic radiotherapy, *TMZ* temozolomide

^a^see Supplemental Material for reference citations

^b^overlapping cases

^c^cases initially diagnosed as epithelioid glioblastoma but clustered with PXA with methylome analysis

The efficacy of therapeutic targeting oncogenically activated kinases in BRAF-mutant cancers depends on structural variations in the kinase domain. For example, the BRAF V600E mutation is often sensitive to kinase inhibitors such as vemurafenib, while β3-αC deletions and non-canonical BRAF mutations are often resistant to this small molecular inhibitor [2]. Therefore, from a therapeutic aspect, it is imperative to define the spectrum of BRAF alterations in these aggressive tumors. Here, we report two newly identified A-PXAs with activating mutations in the β3-αC loop of the BRAF kinase domain discovered through whole-exome, whole-genome, and transcriptome sequencing (Michigan Oncology Sequencing Project [MI-ONCOSEQ]) [8].

The first case is a 5-year-old male presenting with a large (11.7 × 7.3 cm) temporoparietal mass with subfalcine and uncal herniation (Fig. 1a). Molecular profiling revealed an oncogenic BRAF in-frame deletion (p.L485_P490delinsF) located adjacent to the β3-αC loop that results in a helix-constraining conformational change in the kinase domain. The second case is a 23-year-old male with a parietal ring-enhancing cystic mass. Sequencing revealed a novel 9 bp tandem duplication (p.V504_R506dup) in the β3-αC loop that results in a three codon in-frame insertion in the open reading frame (ORF) of BRAF [see Fig. 1a-d and Online Resource for details and representative images from both cases (Additional file 1)]. Consistently, both cases demonstrated MAPK activation with strong expression of phospho-ERK1/2 in tumor cells (Fig. 1g).

**Fig. 1:**
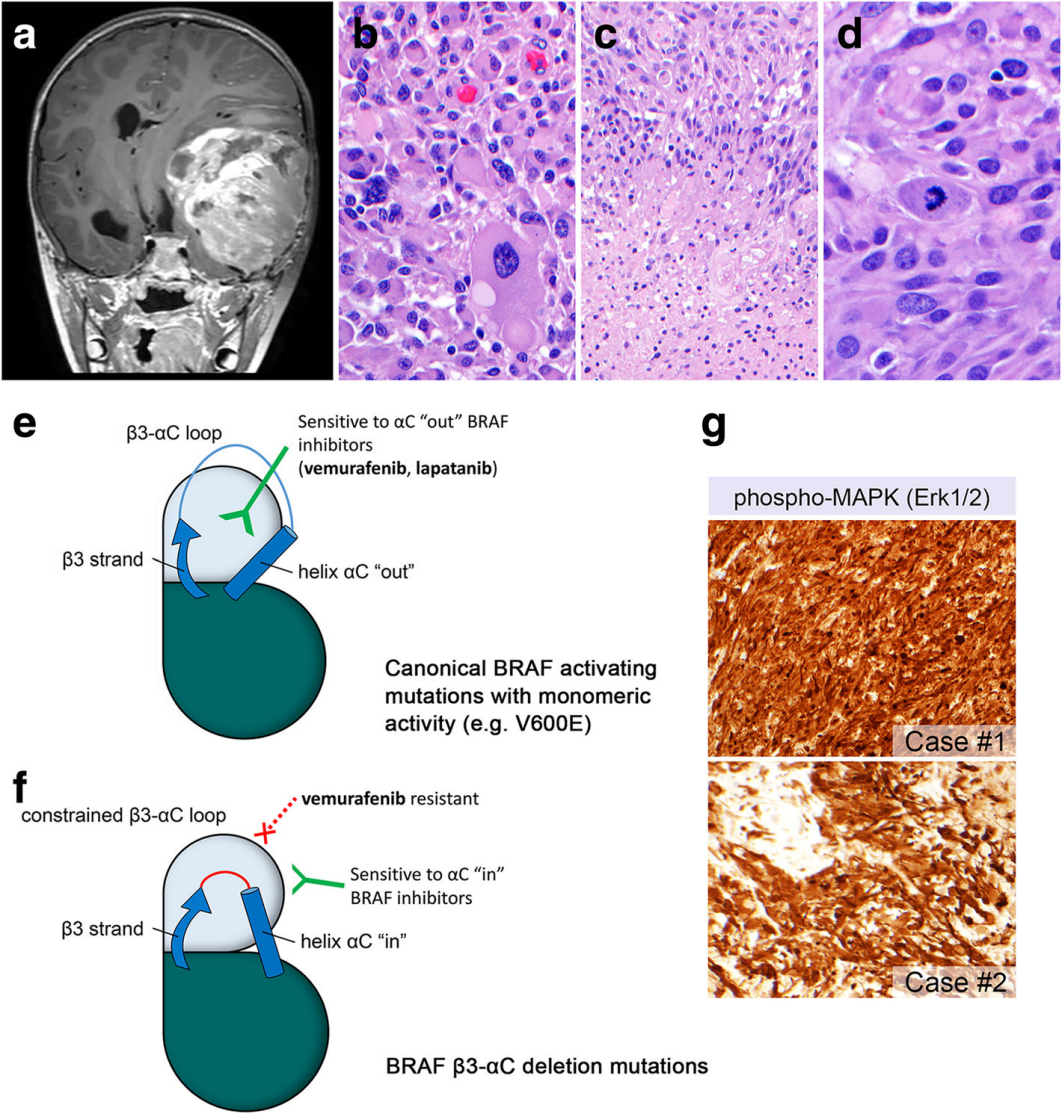
A-PXA with non-V600E activating mutations affecting the β3-αC loop in BRAF. Post-contrast T1-weighted coronal MR sequence showing a large space-occupying lesion with significant midline shift (**a**). Histologic sections from case #1 demonstrated pleomorphic giant cells (**b**), as well as pseudopalisading necrosis (**c**) and increased mitotic activity (**d**). Illustration of conformational changes of the β3 strand and αC helix in the kinase domain. The canonical BRAF V600E mutation results in monomeric activity and can accommodate the oncogenic BRAF inhibitor vemurafenib, which only binds when helix αC is “out” (**e**). In β3-αC deletion mutations, the β3-αC loop is shortened, effectively locking helix αC in the “in” position and conferring resistance to vemurafenib (**f**) (modified with permission from Trends in Cancer, 2 (12), Foster SA, Klijn C, Malek S, Tissue-Specific Mutations in BRAF and EGFR Necessitate Unique Therapeutic Approaches, p. 699–701, 2016, Supplemental Reference [2] [Online Resource]). MAPK signaling pathway activation was confirmed with immunohistochemistry with anti-phospho-p44/42 MAPK [Erk1/2] [Thr202/Tyr204] (**g**)

Both of the mutations reported here affect the β3-αC loop in the kinase domain. To function properly, protein kinases must maintain a level of structural flexibility in order to switch between inactive and active states. This conformational change involves two regulatory regions in the catalytic domain: the activation segment and the αC-helix [2]. During this process, the αC-helix undergoes an “out” to “in” shift that facilitates interaction with the β3 strand and initiates catalysis [2] (Fig. 1e). Case #1 demonstrated a deletion mutation in the BRAF β3-αC loop that results in a shortened αC-helix that constrains the loop conformation to a constitutively kinase active “in” state. Similar “in” state activating alterations have been reported in other major signaling pathway kinases including HER2 and EGFR [2]. β3-αC deletion mutations render tumors resistant to small molecule inhibitors, such as vemurafenib, that bind to and inhibit kinases with an “out” conformation, but are ineffective against the “in” state [1, 2] (Fig. 1e, f). Case #2 contained a mutation in a structural element (R-spine) of the αC-helix [9]. Mutations in the R-spine have been shown to stabilize the active state and result in constitutive kinase activation [3]. However, the effect of this mutation on the conformational state of the kinase domain remains to be determined. Because RAF dimers are often formed in tumors with β3-αC kinase loop alterations, RAF dimer inhibition has been proposed as an alternative therapy for these genetic alterations [10].

Recent reports of clinical responses in V600E-mutated A-PXAs with BRAF “out” inhibitors [5, 7] have been encouraging. However, selection of effective targeted therapies requires a mechanistic understanding of oncogenic kinase activation in tumors. We present two A-PXAs that contain BRAF β3-αC loop alterations that may not be sensitive to traditional BRAF inhibitors. Therefore, treatment approaches for A-PXAs with or without V600E mutations may differ depending on the specific type of BRAF genetic alteration.

## Additional files

Additional file 1: Clinical details, pathologic work-up, and sequencing methodology used in the current study. **Figure S1**. Additional histopathology from case #1 showed characteristic eosinophilic granular bodies (EGBs) (a) and an elevated proliferation index (Ki-67) (b). Immunohistochemistry for p16 showed loss of expression in tumor cells with retained expression in non-neoplastic cells (c, arrowhead), consistent with deletion of the INK4a locus. Staining with mutant-specific BRAF (VE1) was negative (d). **Figure S2**. Case #2 showed lipidized tumor cells and PAS-positive, diastase-resistant EGBs (arrowheads) (a). Nuclear pleomorphism and increased mitotic activity were seen (b, c). Neurofilament stain showing circumscription of the tumor mass (d). BRAF V600E was negative by IHC (e). **Figure S3**. MI-ONCOSEQ integrative sequencing report elements: somatic point mutations for case #1 (a) and #2 (c). Copy number plots for case #1 (b) and #2 (d). (DOCX 13229 kb)
