# Supplementary material for: BRAF activating mutations involving the β3-αC loop in V600E-negative anaplastic pleomorphic xanthoastrocytoma
Source: Acta Neuropathol Commun. 2018 Mar 15;6:24. doi: 10.1186/s40478-018-0525-1 (PMC5855983; doi:10.1186/s40478-018-0525-1)
Supplement: Supplementary file 1 — Clinical details, pathologic work-up, and sequencing methodology used in the current study. Figure S1. Additional histopathology from case #1 showed characteristic eosinophilic granular bodies (EGBs) (a) and an elevated proliferation index (Ki-67) (b). Immunohistochemistry for p16 showed loss of expression in tumor cells with retained expression in non-neoplastic cells (c, arrowhead), consistent with deletion of the INK4a locus. Staining with mutant-specific BRAF (VE1) was negative (d). Figure S2. Case #2 showed lipidized tumor cells and PAS-positive, diastase-resistant EGBs (arrowheads) (a). Nuclear pleomorphism and increased mitotic activity were seen (b, c). Neurofilament stain showing circumscription of the tumor mass (d). BRAF V600E was negative by IHC (e). Figure S3. MI-ONCOSEQ integrative sequencing report elements: somatic point mutations for case #1 (a) and #2 (c). Copy number plots for case #1 (b) and #2 (d). (DOCX 13229 kb) [file 40478_2018_525_MOESM1_ESM.docx]

**Online Resource - Supplemental Material**

**Case Details**

Case #1

A 5-year-old previously healthy male presenting with decreased appetite and daily non-bilious, non-projectile vomiting. MR imaging revealed a large (11.7 x 7.3 x 9 cm) heterogeneous intra-axial solid and cystic mass in the left hemisphere involving the frontal, parietal, and temporal lobes with mass effect (1.6 cm midline shift) and subfalcine and uncal herniation. The solid portions of the tumor showed an enhancing rim without diffusion restriction. There was markedly increased blood volume on perfusion imaging. The patient then underwent two separate partial resections, followed by chemotherapy with the A9952 protocol with carboplatin and vincristine. The patient was alive and stable at last follow-up, 6 months after the initial surgery. Histopathology of the initial resection revealed pleomorphic tumor cells with abundant eosinophilic cytoplasm, cytoplasmic vacuolization, multinucleation, and scattered ganglion-like cells. Eosinophilic granular bodies were noted (**Supp. Fig. 1a**). Increased mitoses and pseudopalisading necrosis were also identified. Immunohistochemistry showed the tumor cells were strongly reactive for GFAP. Neurofilament staining was negative within the tumor. Tumor cells showed retained INI-1 expression. The proliferation index (Ki-67) was focally increased (**Supp. Fig. 1b**). P16 IHC showed loss/decreased expression in tumor cells with retained expression in non-neoplastic cells (**Supp. Fig. 1c**). BRAF V600E staining was negative (**Fig. 1d).**  A diagnosis of anaplastic pleomorphic xanthoastrocytoma, WHO grade III was rendered. BRAF V600E/K was negative by PCR.

Case #2

A 23-year-old male presented with a 6-year history of aura and seizure. Imaging in the ER revealed a right parietal ring-enhancing cystic mass. The patient underwent a subtotal resection and was stable at last follow-up (4 months after diagnosis). Pathology showed a high-grade neoplasm composed of pleomorphic tumor cells with lipidized cytoplasm (**Fig. 2b**), numerous eosinophilic granular bodies (**Fig. 2a**), and a fascicular growth pattern in areas. Numerous mitotic figures (**Fig. 2c**), necrosis, and microvascular proliferation were identified. Neurofilament immunohistochemistry showed localized/circumscribed growth (**Fig. 2d**), and ATRX IHC showed retained nuclear expression. BRAF V600E staining was negative (**Fig. 2e**). The proliferation index (Ki-67) was increased (up to 20% in areas). Molecular testing for IDH1/2 and BRAF V600E was negative. The tumor was diagnosed as anaplastic pleomorphic xanthoastrocytoma, WHO grade III.

**Supplemental Methods**

MI-ONCOSEQ

The Michigan Oncology Sequencing Project (MI-ONCOSEQ) is a multi-platform high-throughput integrated sequencing approach that involves the detection of point mutations, insertions/deletions, amplifications, gene fusions and rearrangements, germline alterations, and gene expression. For detailed methodology, please see Robinson et al.[7].

**Ethics, consent and permissions**

Sequencing studies were performed at the University of Michigan after approval by our Institutional Review Board.

**Supplemental Figure Captions**

Supplemental Fig. 1

Additional histopathology from case #1 showed characteristic eosinophilic granular bodies (EGBs) (**a**) and an elevated proliferation index (Ki-67) (**b**). Immunohistochemistry for p16 showed loss of expression in tumor cells with retained expression in non-neoplastic cells (**c**, arrowhead), consistent with deletion of the *INK4a* locus. Staining with mutant-specific BRAF (VE1) was negative (**d**).

Supplemental Fig. 2

Case #2 showed lipidized tumor cells and PAS-positive, diastase-resistant EGBs (arrowheads) (**a**). Nuclear pleomorphism and increased mitotic activity were seen (**b, c**). Neurofilament stain showing circumscription of the tumor mass (**d**). BRAF V600E was negative by IHC (**e**).

Supplemental Fig. 3

MI-ONCOSEQ integrative sequencing report elements: somatic point mutations for case #1 (**a**) and #2 (**c**). Copy number plots for case #1 (**b**) and #2 (**d**).

Supplemental Fig. 1


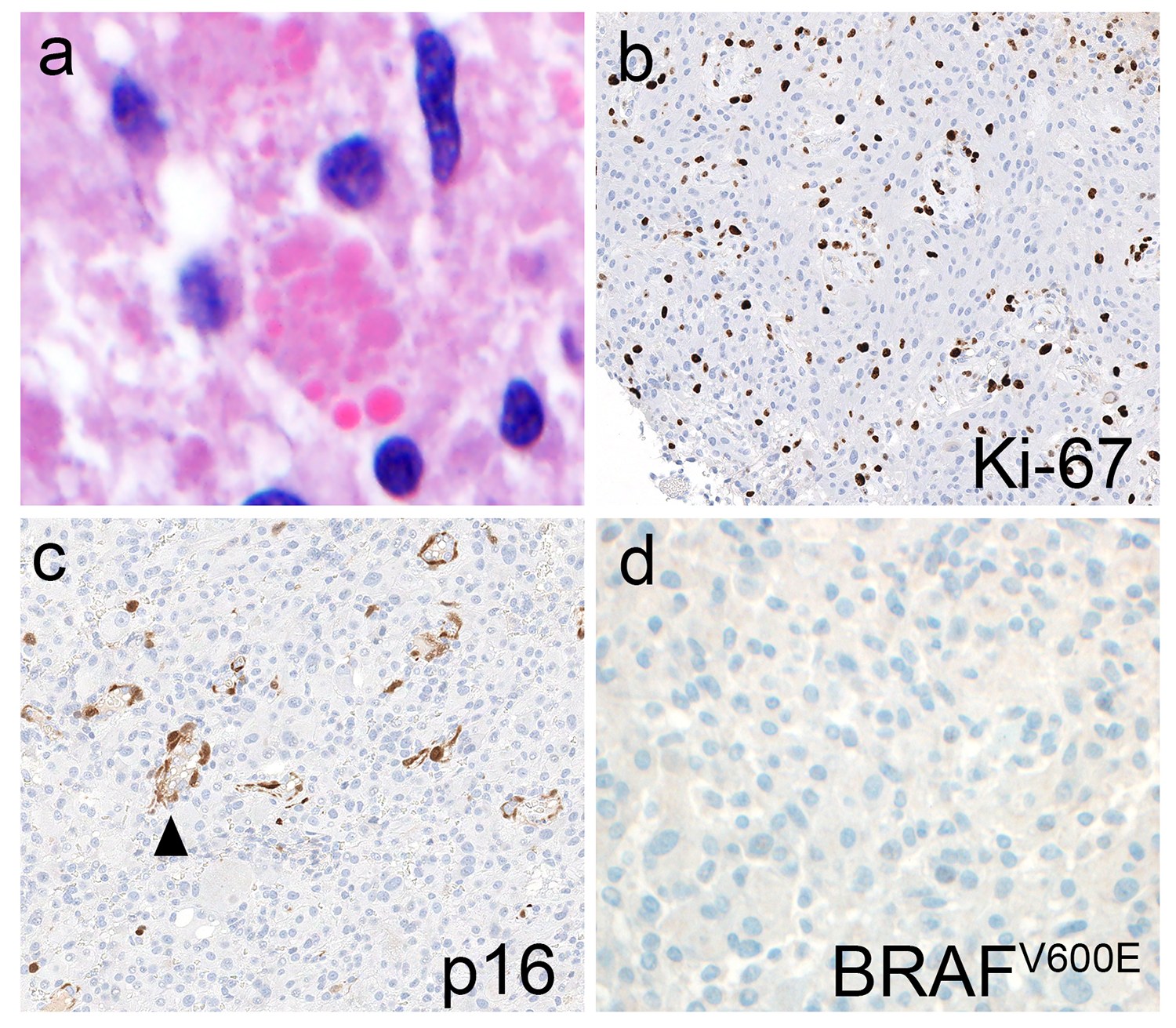


Supplemental Fig. 2


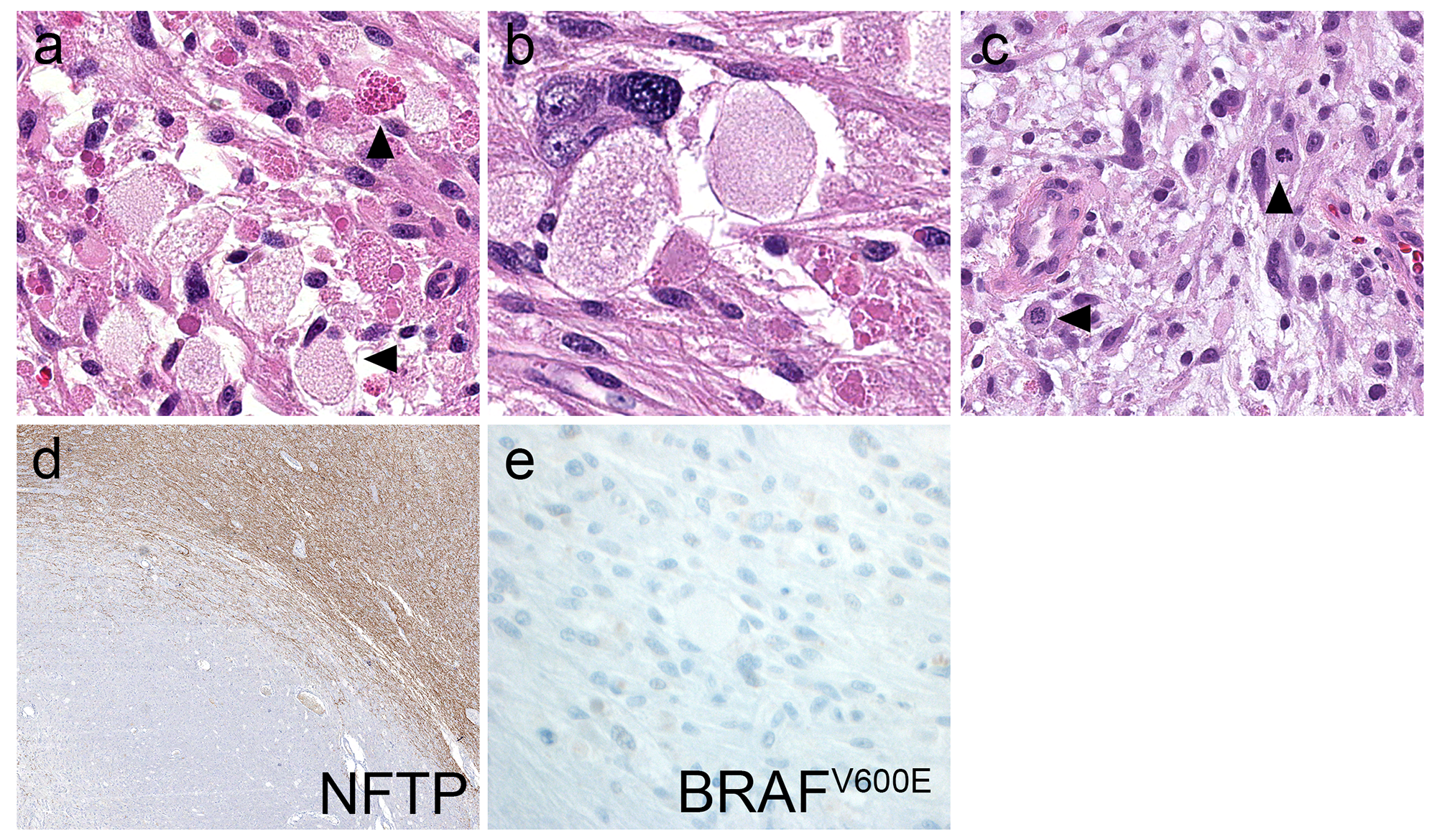


Supplemental Fig. 3


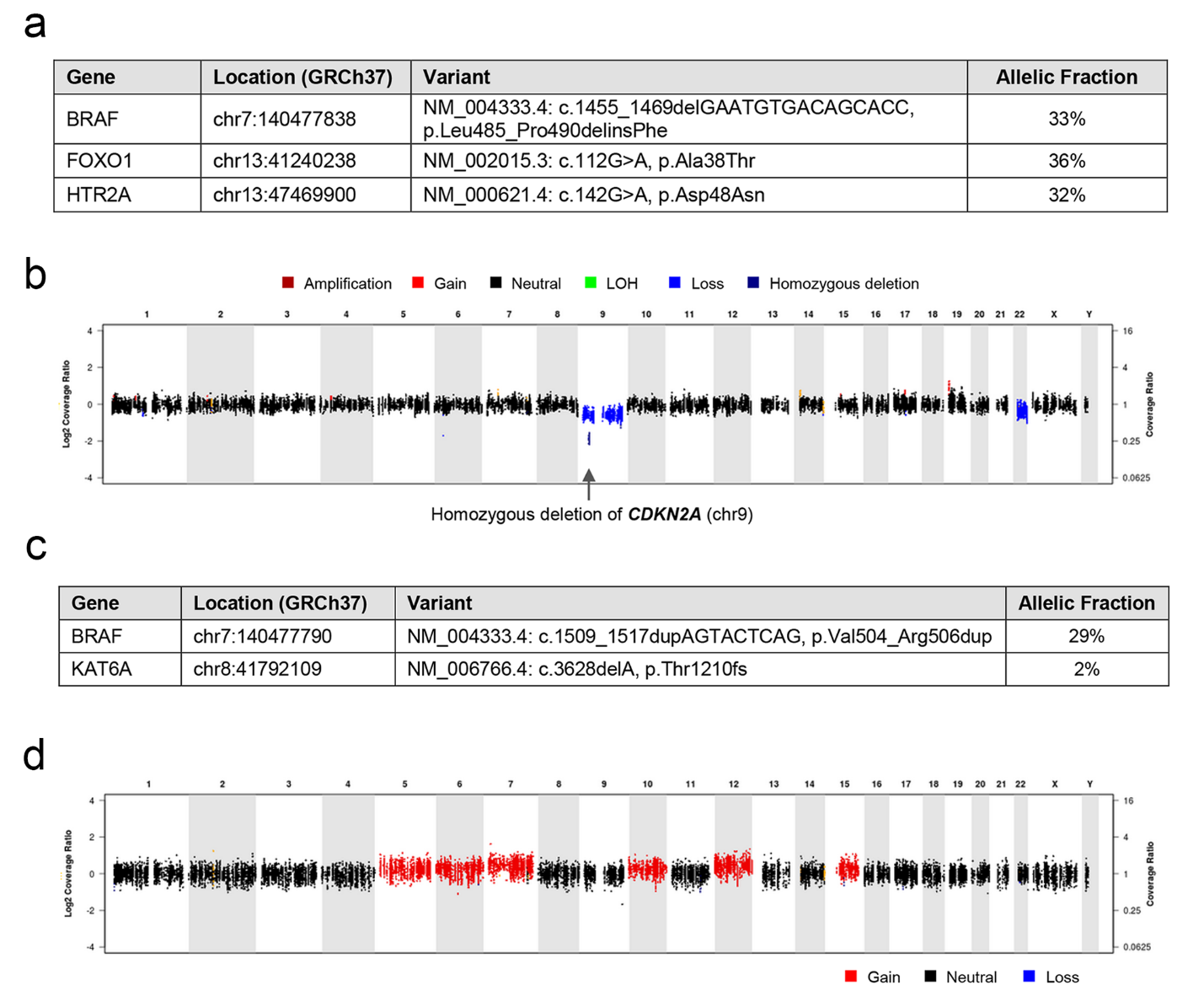


**Supplemental References**

1 Alexandrescu S, Korshunov A, Lai SH, Dabiri S, Patil S, Li R, Shih CS, Bonnin JM, Baker JA, Du Eet al (2016) Epithelioid Glioblastomas and Anaplastic Epithelioid Pleomorphic Xanthoastrocytomas--Same Entity or First Cousins? Brain Pathol 26: 215-223 Doi 10.1111/bpa.12295

2 Foster SA, Klijn C, Malek S (2016) Tissue-Specific Mutations in BRAF and EGFR Necessitate Unique Therapeutic Approaches. Trends Cancer 2: 699-701 Doi 10.1016/j.trecan.2016.10.015

3 Hsiao SJ, Karajannis MA, Diolaiti D, Mansukhani MM, Bender JG, Kung AL, Garvin JH, Jr. (2017) A novel, potentially targetable TMEM106B-BRAF fusion in pleomorphic xanthoastrocytoma. Cold Spring Harb Mol Case Stud 3: a001396 Doi 10.1101/mcs.a001396

4 Korshunov A, Chavez L, Sharma T, Ryzhova M, Schrimpf D, Stichel D, Capper D, Sturm D, Kool M, Habel Aet al (2017) Epithelioid glioblastomas stratify into established diagnostic subsets upon integrated molecular analysis. Brain Pathol: Doi 10.1111/bpa.12566

5 Mistry M, Zhukova N, Merico D, Rakopoulos P, Krishnatry R, Shago M, Stavropoulos J, Alon N, Pole JD, Ray PNet al (2015) BRAF mutation and CDKN2A deletion define a clinically distinct subgroup of childhood secondary high-grade glioma. J Clin Oncol 33: 1015-1022 Doi 10.1200/JCO.2014.58.3922

6 Phillips JJ, Gong H, Chen K, Joseph NM, van Ziffle J, Jin LW, Bastian BC, Bollen AW, Perry A, Nicolaides Tet al (2016) Activating NRF1-BRAF and ATG7-RAF1 fusions in anaplastic pleomorphic xanthoastrocytoma without BRAF p.V600E mutation. Acta Neuropathol 132: 757-760 Doi 10.1007/s00401-016-1616-3

7 Robinson DR, Wu YM, Lonigro RJ, Vats P, Cobain E, Everett J, Cao X, Rabban E, Kumar-Sinha C, Raymond Vet al (2017) Integrative clinical genomics of metastatic cancer. Nature 548: 297-303 Doi 10.1038/nature23306

8 Vaubel RA, Caron AA, Yamada S, Decker PA, Eckel Passow JE, Rodriguez FJ, Nageswara Rao AA, Lachance D, Parney I, Jenkins Ret al (2017) Recurrent copy number alterations in low-grade and anaplastic pleomorphic xanthoastrocytoma with and without BRAF V600E mutation. Brain Pathol: Doi 10.1111/bpa.12495
